# Forensic Psychiatry at a Tertiary Care Hospital in Faisalabad, Pakistan: An Audit From 2015 to 2018

**DOI:** 10.1192/bjo.2024.632

**Published:** 2024-08-01

**Authors:** Irum Siddique

**Affiliations:** ELFT, Bedford, United Kingdom. Department of Psychiatry and Behavioral Sciences, Faisalabad Medical University, Faisalabad, Pakistan

## Abstract

**Aims:**

In Pakistan forensic psychiatry lacks behind as far as formal training and separate departments are concerned. In spite the cases are ever increasing. To find out the magnitude of the burden of forensic cases, current study was conceptualized. This audit would highlight the burden and endorse the demand of specific training in this area. A retrospective study was designed to determine the frequency of various psychiatric disorders, reasons and sources of referrals of the cases coming for forensic opinion to a tertiary care unit.

**Methods:**

All 174 cases admitted to inpatient psychiatry department, Faisalabad for opining about psychiatric condition were included in the study through consecutive sampling techniques, only repeated cases were excluded. As the study was retrospective, data files were retrieved and desired variables were enlisted in SPSS to calculate the frequency and percentage of different variables.

**Results:**

Majority cases were male. One third were referred in year 2018. 47 (27%) criminal cases were being referred while 25 (14.3%) civil cases were received; most of the cases 102 (58.6%) were departmental (cases of the employees of different public departments). As per source of referral 72 (41.3 96%) cases were referred from courts directly, 21 (12.2 96%) cases were directly referred from various departments while most the cases 81 (46.5%) were referred from other public hospitals, As per diagnoses schizophrenia, depression and intellectual disability (ID) were the most prevalent diagnosis with 47 (27%), 41 (23.5%) and 33 (18.9%) cases respectively while 26 (14.9%) cases had no psychiatric diagnosis. 40 (22.9%) cases were advised treatment and follow up, most of these cases 26 (14.9%) were diagnosed as having depression; 30 (17.2%) cases were granted guardianship, 20 (11.4%) out of these were intellectually disabled; 18 (10.2%) cases were referred to other departments for long term psychiatric care institutions, these cases were diagnosed as having schizophrenia, BAD and epilepsy; 9 (5.1%) cases were advised adjustments in jobs, these were diagnosed as depression, schizophrenia and BAD; only 6 (3.4%) cases were suggested to board out on the basis of illness.

**Conclusion:**

Department of psychiatry and behavioral sciences, FUM, Faisalabad, Pakistan is burdened with forensic cases that may be managed at other appropriate places. Society and policy makers need to be sanitized in order to make a framework for patients having mental disorder to avoid them ending as criminals or being involved in other forensic issues.

